# Simultaneous Esophageal and Tracheal Obstructions Caused by a Pair of Magnetic Beads in a Child: A Case Report

**DOI:** 10.3389/fped.2021.765373

**Published:** 2021-11-01

**Authors:** Huan Ren, Dong Shi, Zhaowei Gu, Zhiwei Cao

**Affiliations:** Department of Otolaryngology Head and Neck Surgery, Shengjing Hospital of China Medical University, Shenyang, China

**Keywords:** tracheal foreign body, esophageal foreign body, magnetic foreign body, case report, pediatric

## Abstract

Esophageal and tracheal foreign body ingestion trigger common pediatric emergencies. In this case report, we describe a pediatric patient with simultaneous tracheal and esophageal obstruction caused by foreign bodies. A child aged 2 years and 1 month swallowed a pair of metallic magnetic beads at the same time; one bead entered the trachea and the other bead entered the esophagus. We suspected that the two magnetic beads were mutually attracted and thus became trapped in their respective lumina. The tracheal foreign body was uneventfully removed; this dislodged the esophageal foreign body, which was then excreted. There were no serious complications in the present case, but parents and medical personnel should be mindful of the potential hazards associated with ingestion of multiple magnetic foreign bodies. A high index of suspicion is appropriate. Investigations must be carefully planned. Treatment should not be delayed; the consequences of delay may be serious.

## Introduction

Airway and esophageal foreign bodies are common pediatric emergencies. Airway foreign bodies refer to foreign bodies in the larynx, trachea, and bronchi; the foreign bodies enter primarily through the oral cavity. The location of a foreign body in the airway is generally dependent on the size, shape, and weight of the foreign body; it is also dependent on the patient's position during foreign body ingestion or inhalation, as well as the patient's unique anatomical factors. Most reported cases of airway foreign bodies have involved the right main bronchus.

Esophageal foreign bodies are associated with age, sex, eating habits, an esophageal pathology, consciousness level, and psychiatric status. In children, esophageal foreign bodies are mostly inadvertently swallowed toys; esophageal foreign bodies in older adults are attributable to poor mastication, improper denture use, or ill-fitting dentures. In this case report, we describe the clinical course and successful treatment of concurrent tracheal and esophageal obstruction after the accidental simultaneous swallowing of a pair of magnetic metallic beads by a 2-year-old boy.

## Case Presentation

A boy aged 2 years and 1 month was admitted to our hospital with a 2-week history of wheezing and a 4-day history of labored breathing in mid-December. His family members reported that, 2 weeks prior to admission, he had begun wheezing without any obvious cause; the wheezing was associated with coughing and crying but did not affect his sleep. After he was administered erythromycin, the wheezing did not obviously improve; he then developed labored breathing in the 4 days preceding admission. Chest computed tomography (CT) at the local hospital had revealed a foreign body. Surgical treatment was recommended, and the patient was transferred to our hospital.

On admission, clinical examination revealed no fever, obvious dyspnea, cyanosis, or other remarkable sign. During auscultation, bilateral monophonic wheezing was apparent; this did not improve with changes in body position. After admission, the child was denied food and water; he was rehydrated intravenously in preparation for a possible emergency operation under general anesthesia.

**Figure 1:**
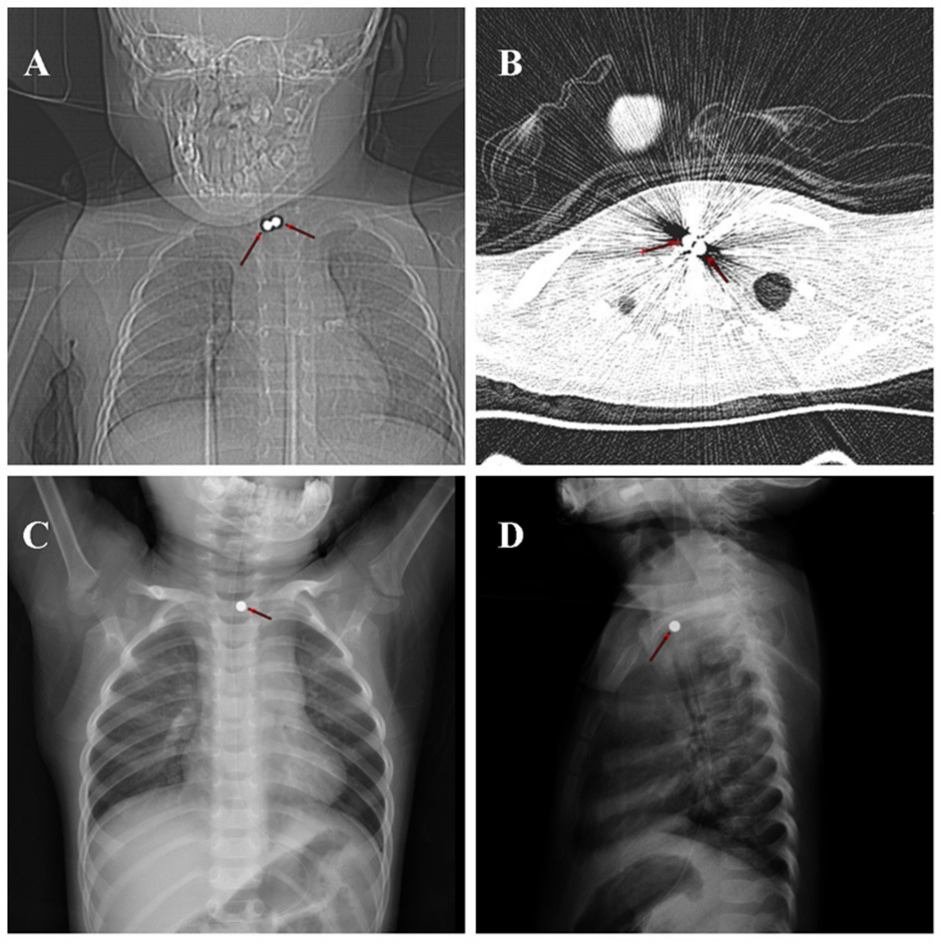
Location mapping and horizontal CT of the trachea and bronchi revealed two round high-density shadows (red arrow) of diameter ~0.5 cm that appeared to be located in the upper part of the trachea at the level of the chest **(A,B)**. A residual round, metallic, foreign body shadow was apparent at the entrance of the thoracic cage on chest frontal and lateral DR performed at 6 h post-operatively **(C,D)**.

Location mapping and horizontal CT of the trachea and bronchi revealed two round high-density shadows of diameter ~0.5 cm that appeared to be located in the middle part of the trachea (~3 cm below the glottis) at the level of the chest. The principal foreign body appeared to be lodged in the tracheal lumen (Figures 1A,B), and a diagnosis of a tracheal foreign body was tentatively made. Foreign body removal (*via* rigid bronchoscopy) was performed with the child under general anesthesia at 8:10 a.m. the following day. During the operation, a round, blue foreign body was found partially obstructing the trachea. The foreign body was aspirated into the bronchoscope and withdrawn. The bronchoscope was re-introduced to confirm that the trachea was unobstructed. After bronchoscope advancement to the bronchi, both bronchial branches were found to be unobstructed. Because imaging had revealed two round high-density shadows, esophagoscopy was performed; thorough exploration revealed no foreign body in the esophagus or pharynx, and the child was returned to the ward.

**Figure 2:**
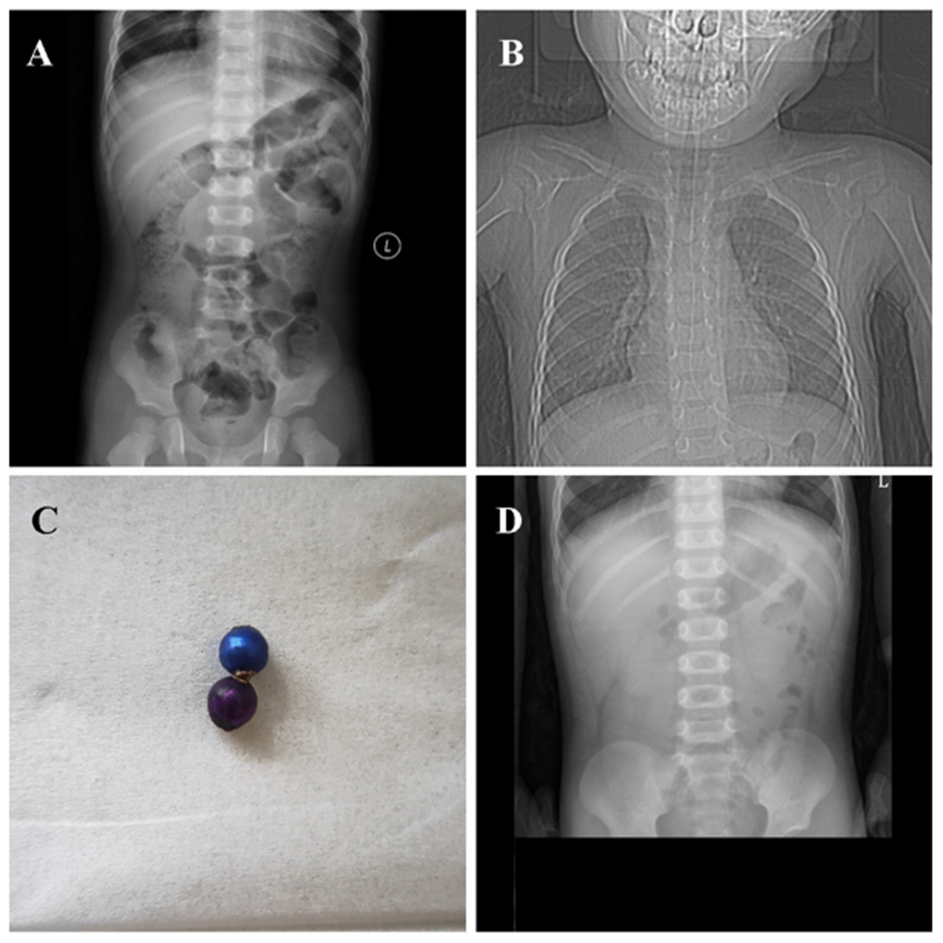
Abdominal DR indicated no obvious abnormality at 6 h post-operatively **(A)**. Chest DR re-examination indicated that the foreign body shadow had disappeared before the planned second operation **(B)**. The blue and purple magnetic beads were magnetically attracted **(C)**. Abdominal DR re-examination indicated no obvious, remaining, foreign body shadow after the second foreign body had been expelled **(D)**.

Post-operative chest and abdominal digital radiography (DR) performed on the same day at 2:35 p.m. revealed a residual round, metallic, foreign body shadow at the entrance of the thoracic cage on chest frontal and lateral DR (Figures 1C,D). The position of this foreign body was identical to the apparent position on pre-operative imaging. Abdominal DR (Figure 2A) indicated no obvious abnormality, and the second foreign body was thus strongly suspected to have lodged in the esophagus. The child did not exhibit a cough, dyspnea, fever, or any other symptom of tracheal obstruction; he also showed no symptom of esophageal foreign body obstruction (e.g., dysphagia or odynophagia). Esophagoscopy (under general anesthesia) was planned for the day after the first operation, but pre-operative chest DR re-examination indicated that the foreign body shadow had disappeared (Figure 2B).

Earlier imaging had revealed that the foreign body was of small volume, with rounded edges, suggesting that spontaneous expulsion from the esophagus (*via* the gastrointestinal tract) was possible. Thus, we instructed the child's family members to monitor his stool for the presence of the foreign body. The child defecated the following morning, and a purple metallic ball similar to the ball removed in the previous operation was found in the stool. The two balls were magnetically attracted (Figure 2C). Abdominal DR re-examination on the same day (Figure 2D) indicated no obvious, remaining, foreign body shadow.

## Discussion

Tracheobronchial foreign bodies are common otolaryngological emergencies, particularly in children. The principal cause of tracheal and esophageal foreign body ingestion in infants is a lack of parental or caregiver supervision. Children lack fully developed teeth; their mastication function and throat reflexes are both immature. Moreover, they tend to place various objects in the mouth while playing, which may trigger tracheal or esophageal occlusion after aspiration or swallowing, respectively. Foreign objects swallowed by children usually include objects commonly found in the home environment, such as coins, toys, jewelry, magnets, and batteries. After foreign body ingestion, the child may show obvious symptoms, such as a dry cough, difficulty breathing, and cyanosis; other manifestations include sudden crying, irritability, food refusal, drooling, or vomiting (1). Because infants and young children cannot verbalize, parents sometimes cannot provide a clear history of foreign body ingestion. If the clinical manifestations are atypical, a foreign body may be missed or misdiagnosed.

The principal symptom in our patient was bilateral monophonic wheezing, audible on auscultation. In spectral terms, wheezes can be divided into monophonic and polyphonic types (2). Polyphonic wheezes usually reflect a pathology of the small airways; monophonic wheezes usually reflect a pathology of the larger airways, and/or asthma (3).

Causes other than asthma must be considered in infants and children with airway obstructions who have shown weak or no response to treatment, as well as previously asymptomatic children who develop sudden or recent wheezing. The most common cause of such a clinical condition is the inhalation of foreign bodies (4). A previous case series reported three cases of foreign body aspiration in pre-schoolers with no history of inhalation: they exhibited only asthma-like symptoms that persisted despite appropriate asthma treatment (5). Other possibilities include acquired tracheal stenosis after endotracheal intubation, lymph node compression of the airways caused by pulmonary tuberculosis and/or a lymphoma, or a lung/large airway malignancy (4). For our patient, chest CT scans from a local hospital were available upon referral to our hospital; an accurate diagnosis was quickly determined.

The location of the foreign body could not be accurately determined because of pre-operative imaging artifacts associated with metallic objects. The child's symptoms and clinical signs caused us to suspect that the foreign body was lodged in the trachea. The foreign body was small, and the child was able to ingest food; hence, we did not suspect an esophageal foreign body. Furthermore, the child had no typical clinical symptom such as food refusal or vomiting. Pre-operative location mapping and horizontal CT of the bronchi indicated that the foreign body was located in the tracheal lumen; imaging did not reveal a foreign body in the esophagus. Therefore, prior to the operation, we suspected that both bodies were lodged in the trachea.

During rigid bronchoscopy, a single magnetic foreign body was removed; we found no other foreign body in the trachea. However, two foreign bodies had been identified pre-operatively; we suspected that the other foreign body may have been in the esophagus. We performed esophagoscopy but found no foreign body. Post-operative re-examination (chest DR) was performed at 6 h post-operatively; the image was compared with pre-operative images. The post-operative image revealed a round, metallic foreign body (~0.5 × 0.5 cm) in the original position. We suspected that the esophageal, metallic foreign body had initially been attracted to the tracheal foreign body through the esophageal mucosa; after the operation, it was embedded in the esophageal wall. During esophagoscopy, the esophagoscope might have been advanced past the foreign body and missed during intraoperative imaging. After removal of the tracheal foreign body, the esophageal foreign body likely remained embedded in the esophageal mucosa and became exposed over time. This allowed the mucosa to relax.

The esophageal foreign body identified by CT was not found during multiple microscopic examinations, indicating that the foreign body had been buried under the mucosal layer. Endoscopic submucosal dissection may be required to locate and remove such a submucosal foreign body (6).

Before the second exploration under general anesthesia, chest DR re-examination revealed no foreign body shadow. Because the previous imaging findings had suggested that the foreign body was small with rounded edges, we expected that it would be excreted naturally (*via* the gastrointestinal tract). Indeed, the child excreted a round, metallic foreign body in the early morning of the second post-operative day. Abdominal DR re-examination confirmed the absence of any foreign body; thus, both foreign bodies had finally been retrieved.

Although tracheal and esophageal foreign bodies are common otolaryngological emergencies, it is generally easy to determine the location of a foreign body based on imaging and clinical symptoms. However, it is rare to encounter foreign bodies that become simultaneously lodged in both the trachea and the esophagus. Because our patient ingested magnetic foreign bodies, ingestion may have been asymptomatic (7). The two foreign bodies (in the trachea and esophagus) were mutually attracted and trapped in fixed positions. Thus, the clinical symptoms differed from the symptoms in patients with isolated, mobile foreign bodies. Patients with typical foreign bodies may present with severe coughing, dyspnea, and/or cyanosis; symptoms of tracheal foreign body obstruction (e.g., stridor); or symptoms of esophageal foreign body obstruction (e.g., food refusal and vomiting). In our patient, the foreign bodies in the trachea and esophagus were mutually attracted and their positions were fixed; thus, there was little change in the patient's condition. In addition, the foreign bodies were small; despite their attraction, the tracheal and esophageal lumina remained open, leading to few of the typical effects associated with tracheal and esophageal foreign body obstruction. An earlier report indicated that, of 85 pediatric patients who ingested magnetic foreign bodies and were admitted to a general surgery department over a 10-year period, almost one-third had no clinical symptoms (8).

The number of cases of magnetic foreign body ingestion has increased in recent years; although safeguards have improved, magnetic toys should be kept out of the reach of toddlers and small children (9). In the United States, the number of emergency department visits related to magnet-related injuries has recently increased by more than three-fold; cases of multiple magnet ingestion significantly increased in number between 1996 and 2012. Magnets used as toys are potentially harmful to both children and adolescents (10). The ingestion of a single magnetic foreign body does not usually cause serious complications; the clinical manifestations are similar to the manifestations of other foreign bodies. However, the ingestion of multiple magnetic foreign bodies can cause more serious clinical manifestations and complications (11).

Radiological investigation is indispensable to confirm the presence of foreign bodies. Although this helps to locate foreign bodies in children, the results are (sometimes) false-positives or false-negatives (12). Two attached magnetic foreign bodies can present as a single foreign body on imaging examination. Therefore, clinicians must study the images carefully prior to surgery. Ingestion of multiple magnets, or a combination of magnets and metal objects, can cause serious complications that require immediate surgical intervention (13). If a foreign body enters the digestive tract, the complications can include intestinal perforation, peritonitis, intestinal fistulation, intestinal volvulus, and small bowel obstruction triggering short bowel syndrome or death (14). The ingestion of multiple magnets can result in a perforation rate of up to 50% (13). The risk of mucosal damage after swallowing magnetic foreign bodies is much higher than the risk of such damage after swallowing other common items. In recent years, there have been many reports from China and abroad concerning the symptoms caused by magnetic beads entering the gastrointestinal tract. After a child accidentally swallows multiple, magnetic foreign bodies, these engage in mutual attraction in the gastrointestinal tract. A greater number of magnets leads to stronger attractive forces. Multiple magnets that successively enter the abdominal cavity may become mutually attracted *via* the intestinal mucosa in varying locations in the intestine. When the magnets become attached, the intervening intestinal tissue is clamped; the magnets cannot separate. This may rapidly trigger the complications mentioned above.

We have described a rare incident involving the accidental ingestion of foreign bodies that became lodged in both the trachea and the esophagus, resulting in trans-mucosal attraction and entrapment in the respective lumina. The various possible anatomical locations of multiple foreign bodies should be carefully considered before surgery, and an optimal surgical plan should be selected to avoid misdiagnosis. Although there were no serious consequences in the present case, medical personnel who encounter patients with signs of foreign body ingestion should maintain a high index of suspicion. It is possible to ingest multiple, magnetic foreign bodies, which cause unique complications. However, this situation is rare.

## Data Availability Statement

The original contributions presented in the study are included in the article/supplementary material, further inquiries can be directed to the corresponding author.

## Ethics Statement

The studies involving human participants were reviewed and approved by Ethics Committee of Shengjing Hospital of China Medical University. Written informed consent to participate in this study was provided by the participants' legal guardian/next of kin.

## Author Contributions

HR, DS, and ZC co-wrote and edited the manuscript. ZG originated idea, co-wrote, and edited the manuscript. All authors contributed to the article and approved the submitted version.

## Conflict of Interest

The authors declare that the research was conducted in the absence of any commercial or financial relationships that could be construed as a potential conflict of interest.

## Publisher's Note

All claims expressed in this article are solely those of the authors and do not necessarily represent those of their affiliated organizations, or those of the publisher, the editors and the reviewers. Any product that may be evaluated in this article, or claim that may be made by its manufacturer, is not guaranteed or endorsed by the publisher.
